# Mortality of the Plague

**Published:** 1843-04-01

**Authors:** 


MORTALITY OF THE PLAGUE.

Of 97 patients treated in the lazzeretto at Alexandria in 1842, 62 were cured, and 35 died. Of the latter, 14 died immediately after the first visit, and 1 during convalescence, from another disease. Of 166 patients treated at their own homes, 151 died, and 15 only recovered.—*Il Filocamo* (Malta Journal.)

